# Determination of hematological and serum biochemical reference values for indigenous sheep (*Ovies aries*) in Dhaka and Chittagong Districts of Bangladesh

**DOI:** 10.14202/vetworld.2018.1089-1093

**Published:** 2018-08-09

**Authors:** Md. Kaisar Rahman, Shariful Islam, Jinnat Ferdous, Md. Helal Uddin, Muhammad Belal Hossain, Mohammad Mahmudul Hassan, Ariful Islam

**Affiliations:** 1 EcoHealth Alliance, NY, USA; 2 Institute of Epidemiology, Disease Control and Research (IEDCR), Dhaka, Bangladesh; 3 Department of Medicine and Surgery, Faculty of Veterinary Medicine, Chittagong Veterinary and Animal Sciences University, Bangladesh; 4 Infectious Disease Division, International Center for Diarrheal Disease Research, Bangladesh; 5 Department of Physiology, Biochemistry, and Pharmacology, Faculty of Veterinary Medicine, Chittagong Veterinary and Animal Sciences University, Bangladesh

**Keywords:** Bangladesh, biochemical parameters, hematology, indigenous sheep, *Ovis aries*, reference values

## Abstract

**Aim:**

The study was aimed to determine the reference values of most commonly used hematological and biochemical parameters of indigenous sheep, reared under semi-intensive backyard farms in Dhaka and Chittagong district, Bangladesh.

**Materials and Methods:**

A total of 41 blood samples were collected from indigenous sheep (*Ovis aries*) from June to December 2016 from Dhaka and Chittagong Districts of Bangladesh. Hematological and serum biochemical parameters such as hemoglobin (Hb), packed cell volume (PCV), erythrocyte sedimentation rate (ESR), total erythrocyte count (TEC), total leukocyte count (TLC), neutrophil, eosinophil, basophil, monocyte, lymphocyte, urea, triglyceride, cholesterol, glucose, albumin, total protein (TP), alanine aminotransferase (ALT), and aspartate transaminase (AST) were determined by biochemical analyzer. 90% reference intervals were calculated for all parameters.

**Results:**

The hematologica and serum biochemical profiles of indigenous sheep showed wide range and variation. The results were categorized according to sex and age of the sheep for comparison. Young sheep had significantly higher PCV, eosinophil, triglyceride, and TP level than that of adult (p<0.05), whereas the urea and albumin level was higher in adult than that of juvenile (p<0.05). Most of the values of the parameters are close to each other for both male and female except TEC, urea, cholesterol, triglyceride, glucose, and AST. However, a significant difference was found only for albumin and basophil level between male and female sheep.

**Conclusion:**

Hematological and biochemical parameters in Bangladeshi indigenous sheep showed a wide range and variation implicating future study for the prophylaxis of ovine diseases.

## Introduction

Small ruminants, especially sheep, are important species for the livestock sector and economic development of Bangladesh. Sheep provide meat and wool and contribute to the total gross domestic product (GDP) of the country [1]. The contribution of the animal farming subsector to GDP at the constant price was 1.78% in 2013-2014. Sheep production has been increased 2.5 times during the past 12 years, and the growth rate was 5% [2]. There are about 3.2 million sheep in Bangladesh, and the number is increasing day by day [1]. Most of the sheep in our country are indigenous. Rural families rear sheep for economic support and for their ability to better adaptation, fertility, good quality meat and skin, wool production and resistance to diseases with low care under adverse agro-climatic condition.

Indigenous native sheep (*Ovis aries*) were originated from wild urial sheep (*O. orientalis vignei*). In Bangladesh, sheep is usually raised on the road and canal side, open uncultivable field with low or minimum care and management [3]. They can easily adjust to hot humid climate and tolerant to diseases [2].

The hematological and biochemical reference values are vital to provide baseline, screening, and diagnosis of diseases [4]. Clinical interpretation of laboratory data of hematology and serum biochemistry is easiest and authentic. Data on hematological and biochemical parameters of indigenous sheep in the Indian subcontinent are limited. Few studies have been done in Indian subcontinent regarding domestic animals such as cattle [5], goat [6], buffalo [7], and small ruminants [8]. However, there is a lack of data related to the reference value for hematology and serum chemistry profiles of indigenous sheep of the Indian subcontinent. A prior study conducted in Bangladesh observed the effect of heat stress on different blood parameters such as red blood cell (RBC), hemoglobin (Hb), white blood cell (WBC), and packed cell volume (PCV) in indigenous sheep [9]. Hematological and biochemical tests are done regularly for sheep, but lack of reference value for these parameters makes it complicated to interpret the results.

Therefore, the present study was aimed to determine the hematological and serum chemistry values of apparently healthy indigenous sheep of Dhaka and Chittagong districts, Bangladesh and the effect of age and gender on the parameters and finally to establish a reference interval (RI) for better prophylaxis and care of ovine health.

## Materials and Methods

### Ethical approval

The Animal Experimentation Ethics Committee of Chittagong Veterinary and Animal Sciences University (CVASU-AEEC) approved the study protocol, and the approval number for the project was CVASU/Dir (R&E) AEEC/2015/927.

### Study design and sample collection

The sheep were selected from some specific regions of Dhaka (n=29) and Chittagong (n=12) Districts of Bangladesh during June to December 2016 those were convenient to collect sample. The average temperature and humidity of the study area of Dhaka were 31°C in June and 20°C in December. The humidity varies from 96% (June) to 100% (December) with low clouds. In Chittagong, the temperature varies from 30°C in June to 22°C in December with an average humidity of 71-72%. Two categories of sheep were formed for sample collection - adult (>6 months of age) and juvenile (<6 months of age).

Approximately 5 mL of blood samples were collected from the jugular vein of each animal, and from there, 3 mL was transferred to a sterile vial containing ethylene diamine tetra-acetic acid (1 mg/mL of blood) for hematological parameters such as Hb, PCV, erythrocyte sedimentation rate (ESR), total leukocyte count (TLC), total erythrocyte count (TEC), using the method described previously by Hossan *et al*. [6], and rest 2 mL was transferred to vacutainer for serum preparation. Centrifugation was done at 3000 rpm for 15 min for serum separation. Then, the serum was transferred to Eppendorf tube for storing at −20°C. All samples were processed at the laboratory of the Departments of Physiology, Biochemistry, and Pharmacology of Chittagong Veterinary and Animal Sciences University (CVASU). Serum samples were tested using automatic biochemical analyzer (Humalyzer-3000, USA) to estimate urea, triglyceride, cholesterol, albumin, glucose, total protein (TP), alanine aminotransferase (ALT), and aspartate transaminase (AST).

### Statistical analysis

Data were stored in Microsoft Excel 2007 (Microsoft Corporation, Redmond, WA 98052-6399 USA) and then exported to MedCalc Statistical Software version 17.5.5 (MedCalc Software bvba, Ostend, Belgium; http://www.medcalc.org; 2017) for estimating mean, standard deviation (SD), and RIs. The robust method was used to calculate RIs because the number of samples collected was 40≤x≥120 (x=41) [10]. The 90% confidence intervals (CI) were calculated for each RI using the bootstrap method. Student’s t-test was used for the comparison of mean values among the different age groups and sex. A significance level of p<0.05 was used.

## Results

Among the sampled sheep, 26 (63%) were adult, and 15 (37%) were juvenile, whereas 20 (48.8%) were male and 21 (51.2%) were female. The hematology and serum chemistry values (mean, SD, minimum, maximum, and RI - 90% CI) of indigenous sheep have been listed in Table-1. Most of the values showed a wider range of RI.

**Table-1:** Biochemical and hematological profiles of indigenous sheep *(O. aries)*, Bangladesh (n=41), with RI.

Parameter	Mean±SD	Min	Max	RI lower limit (90% CI)	RI upper limit (90% CI)
HB (g/dl)	10.15±0.83	8.5	11.8	8.52 (8.1/8.9)	11.78 (11.4/12.2)
PCV (%)	30.56±3.66	22	38	23.38 (21.7/25.0)	37.74 (36.1/39.4)
ESR (mm/h)	0.57±0.38	0	1	-0.17 (-.34/0.001)	1.31 (1.2/1.5)
TLC (thousand/cumm)	7.1±1.22	4.7	9.7	4.7 (4.2/5.3)	9.5 (8.9/10.1)
TEC (million/cumm)	7.3±1.4	4.6	9.8	4.5 (3.9/5.2)	9.9 (9.3/10.5)
Lymphocyte (%)	55.93±5.82	48	68	44.53 (41.9/47.1)	67.32 (64.7/69.9)
Neutrophil (%)	34.63±6.09	24	43	22.69 (19.9/25.4)	46.58 (43.8/49.3)
Eosinophil (%)	4.51±1.50	1	8	1.57 (0.9/2.2)	7.46 (6.8/8.1)
Monocyte (%)	2.68±0.98	1	4	0.75 (0.3/1.2)	4.62 (4.2/5.1)
Basophil (%)	0.49±0.50	0	1	-0.50 (-0.7/-0.3)	1.48 (1.3/1.7)
Urea (mg/dl)	21.47±7.22	6.5	35.1	7.32 (4.1/10.6)	35.62 (32.4/38.9)
Triglyceride (mg/dl)	24.22±6.18	12.2	41.1	12.11 (9.3/14.9)	36.33 (33.6/39.1)
Cholesterol (mg/dl)	45.70±11.13	29.8	69.8	23.89 (18.9/28.9)	67.52 (62.5/72.5)
Albumin (mg/dl)	3.0±0.60	1.57	4.05	1.82 (1.6/2.1)	4.19 (3.9/4.5)
Glucose (mg/dl)	39.92±15.17	11.7	74	10.19 (3.4/16.9)	69.66 (62.9/76.5)
TP (g/dl)	6.61±1.26	4.37	9.54	4.14 (3.6/4.7)	9.1 (8.5/9.6)
ALT (U/L)	23.4±7.7	10.4	38.4	8.4 (5.0/11.9)	38.4 (34.9/41.8)
AST (U/L)	99.46±26.09	55.1	147.7	48.32 (36.6/60.0)	150.6 (138.9/162.3)

SD=Standard deviation, Min=Minimum, Max=Maximum, RI=Reference interval, CI=Confidence interval, Hb=Hemoglobin, PCV=Packed cell volume, ESR=Erythrocyte sedimentation rate, TLC=Total leukocyte count, TEC=Total erythrocyte count, TP=Total protein, ALT=Alanine aminotransferase, AST=Aspartate transaminase, *O. aries: Ovisaries*

Considering the difference in age and sex, the level of hematological and serum chemistry parameters differ significantly between adult and juvenile and between male and female indigenous sheep, respectively. Hb (p=0.048), PCV (p=0.03), eosinophil (p=0.001), urea (p=0.01), triglyceride (p=0.006), albumin (p=0.003), and TP (p=0.04) values showed significant difference between juvenile and adult sheep. Young sheep have significantly higher PCV, eosinophil, triglyceride, and TP level than that of adult (p<0.05), whereas urea and albumin level was higher in adult than juvenile (p<0.05) (Table-2).

**Table-2:** Comparison of biochemistry and hematology between healthy adult and juvenile indigenous sheep *(O. aries)* of Bangladesh.

Parameters	Category (n)	Mean±SD	95% CI	p-value
Hb (g/dl)	Adult (26)	9.94±0.74	9.6-10.2	0.048
	Juvenile (15)	10.5±0.23	10.01-10.9	
PCV (%)	Adult (26)	29.6±3.4	28.2-30.9	0.03
	Juvenile (15)	32.2±3.5	30.2-34.2	
ESR (mm/h)	Adult (26)	0.6±0.41	0.45-0.78	0.33
	Juvenile (15)	0.5±0.33	0.31-0.68	
TLC (thousand/cumm)	Adult (26)	6.9±1.09	6.5-7.4	0.50
	Juvenile (15)	7.3±1.4	6.5-8.1	
TEC (million/cumm)	Adult (26)	7.5±1.4	6.9-8.08	0.12
	Juvenile (15)	6.8±1.2	6.1-7.5	
Lymphocyte (%)	Adult (26)	55.4±6.2	52.9-57.9	0.44
	Juvenile (15)	56.8±5.1	53.9-59.6	
Neutrophil (%)	Adult (26)	35.2±6.3	32.7-37.7	0.44
	Juvenile (15)	33.7±5.9	30.4-36.9	
Eosinophil (%)	Adult (26)	4±1.5	3.4-4.6	0.001
	Juvenile (15)	5.4±0.9	4.8-5.9	
Monocyte (%)	Adult (26)	2.6±1.1	2.2-3.1	0.79
	Juvenile (15)	2.7±0.8	2.3-3.2	
Basophil (%)	Adult (26)	0.5±0.5	0.2-90.7	0.84
	Juvenile (15)	0.46±0.1	0.18-0.7	
Urea (mg/dl)	Adult (26)	23.8±5.7	21.5-26.1	0.01
	Juvenile (15)	17.4±7.9	13-21.77	
Triglyceride (mg/dl)	Adult (26)	22.2±5.49	19.9-24.4	0.006
	Juvenile (15)	27.7±5.89	24.4-30.9	
Cholesterol (mg/dl)	Adult (26)	45.7±10.2	41.6-49.8	0.99
	Juvenile (15)	45.7±3.3	38.5-52.8	
Albumin (mg/dl)	Adult (26)	3.2±0.5	2.9-3.4	0.003
	Juvenile (15)	2.6±0.5	2.4-2.9	
Glucose (mg/dl)	Adult (26)	36.9±14.8	30.9-42.9	0.09
	Juvenile (15)	45.1±14.9	36.9-53.4	
TP (g/dl)	Adult (26)	6.3±1.2	5.8-6.8	0.04
	Juvenile (15)	7.1±1.2	6.57.8	
ALT (U/L)	Adult (26)	22.4±7.7	19.3-25.6	0.27
	Juvenile (15)	25.2±7.6	21.1-29.3	
AST (U/L)	Adult (26)	102.2±30.2	90.02-114.4	0.3
	Juvenile (15)	94.7±16.6	85.5-103.8	

SD=Standard deviation, CI=Confidence interval, Hb=Hemoglobin, PCV=Packed cell volume, ESR=Erythrocyte sedimentation rate, TLC=Total leukocyte count, TEC=Total erythrocyte count, TP=Total protein, ALT=Alanine aminotransferase, AST=Aspartate transaminase, *O. aries: Ovisaries*

The TEC, urea, cholesterol, triglyceride, glucose, and AST level showed close values between male and female sheep. However, a significant difference has been found only for albumin and basophil level between male and female sheep (p<0.05) (Table-3).

**Table-3:** Comparison of biochemistry and hematology profiles between healthy male and female indigenous sheep *(O. aries)* of Bangladesh.

Parameters	Sex group (n)	Mean±SD	95% CI	p-value
Hb (g/dl)	Male (20)	10.1±0.9	9.610.5	0.66
	Female (21)	10.2±0.6	9.8-10.5	
PCV (%)	Male (20)	30.9±3.7	29.2-32.7	0.56
	Female (21)	30.2±3.7	28.5-31.9	
ESR (mm/h)	Male (20)	0.5±0.4	0.3-0.7	0.23
	Female (21)	0.6±3.9	0.5-0.8	
TLC (thousand/cumm)	Male (20)	7±1.3	6.4-7.7	0.71
	Female (21)	7.2±1.1	6.7-7.7	
TEC (million/cumm)	Male (20)	7.6±1.2	7-8.2	0.11
	Female (21)	6.9±1.4	6.2-7.6	
Lymphocyte (%)	Male (20)	55.3±5.8	52.7-58.1	0.54
	Female (21)	56.4±5.9	53.8-59.1	
Neutrophil (%)	Male (20)	34.3±5.7	31.7-36.9	0.77
	Female (21)	34.9±6.7	31.9-37.9	
Eosinophil (%)	Male (20)	4.8±1.1	4.3-5.3	0.23
	Female (21)	4.2±1.9	3.4-5.1	
Monocyte (%)	Male (20)	2.9±0.9	2.4-3.4	0.17
	Female (21)	2.5±1.1	2.0-2.9	
Basophil (%)	Male (20)	0.7±0.5	0.5-0.9	0.01
	Female (21)	0.3±0.5	0.7-0.5	
Urea (mg/dl)	Male (20)	20.1±6.5	17.1-23.2	0.24
	Female (21)	22.8±7.8	19.2-26.3	
Triglyceride (mg/dl)	Male (20)	23.7±5.3	21.2-26.2	0.59
	Female (21)	24.8±7.1	21.6-27.9	
Cholesterol (mg/dl)	Male (20)	43.8±10.9	38.8-48.9	0.30
	Female (21)	47.4±11.3	42.2-52.7	
Albumin (mg/dl)	Male (20)	2.8±0.6	2.6-3.1	0.09
	Female (21)	3.1±0.7	2.8-3.4	
Glucose (mg/dl)	Male (20)	38.8±14.8	31.8-45.7	0.62
	Female (21)	41.1±15.9	33.8-48.3	
TP (g/dl)	Male (20)	6.2±0.9	5.9-6.7	0.09
	Female (21)	6.9±1.5	6.2-7.7	
ALT (U/L)	Male (20)	23.3±7.3	19.9-26.8	0.96
	Female (21)	23.4±8.2	19.8-27.3	
AST (U/L)	Male (20)	102.7±26.8	90.2-115.2	0.44
	Female (21)	96.4±25.7	84.7-108.1	

SD=Standard deviation, CI=Confidence interval, Hb=Hemoglobin, PCV=Packed cell volume, ESR=Erythrocyte sedimentation rate, TLC=Total leukocyte count, TEC=Total erythrocyte count, TP=Total protein, ALT=Alanine aminotransferase, AST=Aspartate transaminase, *O. aries: Ovisaries*

## Discussion

The present study was aimed to determine the RI values for important hematological and biochemical parameters for indigenous sheep of Bangladesh. We found a low level of Hb and glucose in indigenous sheep than free-ranging desert bighorn sheep (*O. canadensis*) [11] and domestic sheep [12], whereas Awassi ewes in Jordan [13] had almost similar Hb level to our indigenous sheep. Domestic sheep of Alaska also has higher Hb level than that of sampled sheep [14]. However, albumin and TP level of studied sheep was almost similar to that of desert bighorn sheep [11]. Captive bighorn sheep and wild bighorn sheep have higher values for Hb, PCV, glucose, cholesterol, and TP than that of Bangladeshi indigenous sheep [15]. Cholesterol level in Awassi sheep (87.0±3.4) [13] and Coimbatore sheep (81.8±5.2) was much higher than that of indigenous sheep. This variation can be influenced by sex, breed, physiological status, geographical area, etc.

Sheep from Kashmir region showed almost the same values as indigenous sheep for Hb and AST but upper values for glucose, cholesterol, and ALT [8]. Indigenous sheep have lower AST level than other sheep studied till date [11]. ALT level has been observed higher in our study than West African sheep (10.0±1.1 U/L) [16] but lower than wild sheep (29.15±3.2 U/L) from Iran [17]. There is a variation in ALT level among different types of sheep with different geographical distributions. The ALT level and AST level for sheep were estimated as 5-18 and 75-339 U/L, respectively, by the University of California, Davis. However, the range for ALT was much higher and for AST was much lesser in this study. However, glucose, cholesterol, TP, and albumin level agree to the level stated by UC Davis.

Lymphocyte and monocyte count was found higher in studied sheep than wild Dall’s sheep (*O. dalli dalli*) but lower eosinophil count and Hb level than Dall’s sheep. Comparing to Nelson’s bighorn sheep (*O. canadensis nelsoni*), lymphocyte and eosinophil level is higher, but monocyte level was lower in our sheep. Domestic sheep of Alaska has higher lymphocyte and basophil count than our indigenous sheep [14]. The variation in WBC count may be due to altitude, infection status, production stage, nutritional status, etc.

The RI values were also categorized for different age and sex group of the animal to compare the statistical significance. Juvenile sheep have greater RBC mass than adults measured by high Hb and PCV level. Higher RBC mass was also found for young bighorn sheep (0-2 years) earlier [18]. This difference may be attributable to higher body metabolism of young animals. Younger sheep have significantly higher eosinophil count also. Any parasitic infection can cause an increased number of eosinophil in blood [12].

Urea is a product of protein metabolism which was found in adult sheep at a high level than younger. This is may be due to increased protein intake with the increased age of animals. Moreover, developing immune system results in lower protein in blood level [19]. A prior study reported low protein concentration in young bighorn sheep and low blood urea nitrogen (BUN) in older bighorn ram [18]. Low BUN was also found for free-ranging desert bighorn sheep [11].

Although triglyceride and cholesterol level should be related to each other, but triglyceride level was found higher in young sheep, and cholesterol level was almost similar in two age groups. There may be higher body fat in young sheep which was sampled for the study and this resulted in higher triglyceride level.

Higher basophil count was found in male than the female sheep. Allergic condition sometimes influences the increased number of basophil. The male sheep that were sampled may suffer from any kind of allergic reaction at that time. Sometimes, the stress of animal resulted in the release of corticosteroid or epinephrine and increased the number of WBC count. Albumin level was higher in female than that of male. All these variations in different parameters may be due to diet, water level of body, stress, hormonal influence, and surrounding environmental condition. No significant association has been found between age, sex, and these parameters [8].

## Conclusion

There is extensive variation in the serum biochemical and hematological reference values for indigenous sheep compared to other sheep species. The variation was not influenced by age and sex. So this variation can be attributed by strain, breed, physiological status, geographical condition, temperature, humidity, etc. The present study tried to give a baseline reference range for hematological and biochemical parameters which help for better prophylaxis of ovine health in this subcontinent in future.

## Authors’ Contributions

AI initiated and planned the research; MKR and JF made literature review and data analysis; SI and MHU were involved in the sample collection and data analysis. SI, MBH, MMH, and AI made the necessary corrections over the entire manuscript. All authors read and approved the final manuscript.
